# Amelioration of Cigarette Smoke-Induced Mucus Hypersecretion and Viscosity by *Dendrobium officinale* Polysaccharides *In Vitro* and *In Vivo*

**DOI:** 10.1155/2020/8217642

**Published:** 2020-10-21

**Authors:** Rui Chen, Yingmin Liang, Mary Sau Man Ip, Kalin Yanbo Zhang, Judith Choi Wo Mak

**Affiliations:** ^1^Department of Medicine, The University of Hong Kong, Hong Kong; ^2^School of Chinese Medicine, Li Ka Shing Faculty of Medicine, The University of Hong Kong, Hong Kong; ^3^Department of Pharmacology & Pharmacy, The University of Hong Kong, Hong Kong

## Abstract

Chronic obstructive pulmonary disease (COPD), characterized by oxidative stress and inflammation, is one of the leading causes of death worldwide, in which cigarette smoke (CS) is the major risk factor. *Dendrobium officinale* polysaccharides (DOPs) are the main active ingredients extracted from *Dendrobium officinale*, which have been reported to have antioxidant and anti-inflammatory activity as well as inhibition of mucin gene expression. This study is aimed at investigating the effect of DOPs on CS-induced mucus hypersecretion and viscosity *in vitro* and *in vivo*. For *in vitro* study, primary normal human bronchial epithelial cells (HBECs) differentiated at the air-liquid interface (ALI) culture for 28 days were stimulated with cigarette smoke medium (CSM) in the absence or presence of various concentrations of DOPs or N-acetylcysteine (NAC) for 24 hours. For *in vivo* study, male Sprague-Dawley rats were randomized to sham air (SA) as control group or CS group for 56 days. At day 29, rats were subdivided and given water as control, DOPs, or NAC as positive control as a mucolytic drug via oral gavage for the remaining duration. Samples collected from apical washing, cell lysates, bronchoalveolar lavage (BAL), and lung tissues were evaluated for mucin gene expression, mucus secretion, and viscosity. DOPs ameliorated the CS-induced mucus hypersecretion and viscosity as shown by the downregulation of MUC5AC mRNA, MUC5AC secretary protein, and mucus viscosity via inhibition of mucus secretory granules in both *in vitro* and *in vivo* models. DOPs produced its effective effects on the CS-induced mucus hypersecretion and viscosity via the inhibition of the mucus secretory granules. These findings could be a starting point for considering the potential role of DOPs in the management of the smoking-mediated COPD. However, further research is needed.

## 1. Introduction

Chronic obstructive pulmonary disease (COPD), which is characterized by persistent airflow limitation and airway inflammation, is the third leading cause of death globally [1]. Cigarette smoke (CS), as the major risk factor for COPD, has been reported to be associated with chronic airway inflammation, airway epithelium impairment, and mucus hypersecretion [2–4]. Traditional pathogenetic theory is linking COPD with inflammatory process; however, recent finding revealed the significant role of chronic mucus hypersecretion in the pathogenesis of COPD [5]. Therefore, new treatment should be developed targeting on mucus hypersecretion.

The airway epithelial surface is covered by mucus, which is an extracellular gel with two major components, water and mucins, protecting the lung from continuous environmental exposure [6]. Under healthy condition, the sputum mucus solid concentration is 1.7% (by weight), which is increased to 3.7% in COPD patients [7]. The viscosity of mucus is determined by mucus solid concentration, increased mucus viscosity led to disability of cilia clearance [8]. Mucus is mainly secreted by epithelial surface goblet cells and secretory cells from submucosal glands in large airways [6]. Mucin-5AC (MUC5AC) is the major gel-forming mucin in proximal airways by surface goblet cells, which are the predominant subtype found in COPD patients [9]. Moreover, CS could also induce MUC5AC mucin overexpression, and the increased MUC5AC is correlated with smoking history [3, 10]. Epidermal growth factor receptor (EGFR) might play a crucial role in the regulatory mechanism of CS-induced mucus hypersecretion [11]. Cigarette smoke activates EGFRs which activate mitogen-activated protein (MAP) kinases and cause upregulation of mucin MUC5AC in airway epithelial cells and led to mucus hypersecretion [12]. Due to the increased synthesis and secretion of mucins, mucus is usually dehydrated and more viscous, impeding mucus clearance in COPD airways [13]. N-Acetylcysteine (NAC) has been widely used as a mucolytic drug; however, its efficacy is limited [14]. Therefore, more effective mucolytic drugs should be developed.

The genus *Dendrobium* is one of the largest groups of the family *Orchidaceae*. In China, more than fifty *Dendrobium*-based health food products for promoting body fluid production have been approved by the State Food and Drug Administration (see Supplementary Table (available here)). Polysaccharides are considered as one of the main active ingredients *in Dendrobium* plants [15]. *Dendrobium officinale* polysaccharides (DOPs) have been reported to possess multiple pharmacological activities including antioxidant, anti-inflammatory, antiapoptotic, and hypoglycemic activities [16]. Recent findings demonstrated that DOPs inhibited MUC5AC expression, leading to amelioration of airway inflammation in a rat model of COPD as well as improvement of vital capacity in COPD patients [17]. However, insight into the mechanism of DOPs on inhibiting the CS-indusssssced MUC5AC overproduction in relation to viscosity of mucus in the airways is lacking. This study is aimed at investigating the effects of DOPs on CS-induced mucus hypersecretion and viscosity.

## 2. Materials and Methods

### 2.1. Cigarette Smoke Medium (CSM) Preparation

Cigarette smoke (CS) generated from two cigarettes in the same packet with the mouthpiece filters removed (Camel (11 mg tar, 0.8 mg nicotine); R.J. Reynolds, Winston-Salem, NC, USA) was drawn into a syringe before bubbling in 20 ml phosphate-buffered saline (PBS). The solution was filter-sterilized through a 0.22 *μ*m membrane filter and regarded as 100% CSM. The CSM was standardized by measuring absorbance (OD = 1.1) at 320 nM wavelength using a spectrophotometer CLARIOstar (BMG Labtech; Ortenberg, Germany) and was stored in aliquots at -80°C.

### 2.2. Preparation of DOPs

DOPs were extracted by Hong Kong GMP Pharmaceutical Factory (Bright Future Pharmaceutical Laboratories Ltd.). High-performance liquid chromatography (HPLC) was performed to fingerprint total DOPs (see Supplementary Figure).

### 2.3. DOP Treatment in CSM-Exposed Air-Liquid Interface (ALI) Culture of Primary Human Bronchial Epithelial Cells

**Figure 1:**
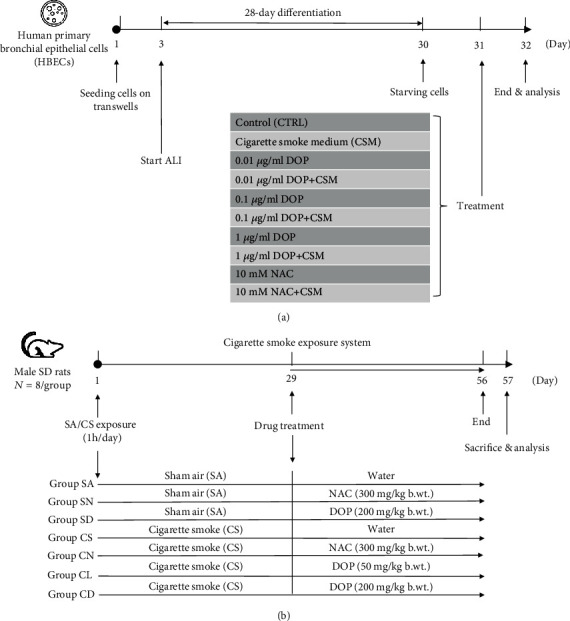
Experimental protocols for *in vitro* and *in vivo* models. (a) Schematic overview of air-liquid interface (ALI) culture of well-differentiated HBEC model with different treatments (*n* = 5). (b) Schematic overview of male Sprague-Dawley (SD) rat model with different treatment groups (*n* = 8).

Well-differentiated normal primary human bronchial epithelial cells (HBECs; *n* = 5) were prepared using a previously described protocol [18]. HBECs from 2 different donors (Lonza, Walkersville, MD, USA; American Type Culture Collection (ATCC), Rockville, MD, USA) were seeded onto collagen-coated 12 mm transwell inserts with 0.4 *μ*m pore size (2.5 × 10^5^ cells per well; Corning Life Sciences, MA). After 100% confluent, medium was removed from the apical side of transwell and left to 28-day differentiation in an ALI medium with 1 : 1 mixture of BEBM (Lonza, Walkersville, MD, USA) and DMEM (Gibco, Carlsbad, CA) supplemented with 52 *μ*g/ml bovine pituitary extract (BPE), 5 *μ*g/ml insulin, 0.5 *μ*g/ml hydrocortisone, 10 *μ*g/ml transferrin, 0.5 *μ*g/ml epinephrine, 0.5 ng/ml human epidermal growth factor (hEGF), 1.5 *μ*g/ml bovine serum albumin (BSA), and 15 ng/ml retinoic acid. After 18 h starvation, cells were treated with 4% CSM in the absence or presence of various concentrations of DOPs (0.01, 0.1, or 1 *μ*g/ml) or N-acetylcysteine (NAC; 10 nM, as positive control) for 24 hours (Figure 1(a)). Apical washing (350 *μ*l) from ALI cultures of well-differentiated HBECs was collected and frozen in aliquots for further analysis.

### 2.4. DOP Treatment in CS-Exposed Rats

Equal numbers (*n* = 8) of male Sprague-Dawley (SD) rats were randomly divided into sham air (SA) as control group or CS group for 56 days. Rats in the CS group were exposed to CS using the computer-controlled whole body inExpose smoking system (SCIREQ, Montreal, Canada) at a total particulate matter (TPM) of 2000 mg/m^3^ for 1 hour (20 cigarettes) daily, while rats in the SA group were subjected to the same procedure in another ventilated chamber but exposed to fresh air. Cigarettes (10 mg TAR and 0.8 mg nicotine, Camel, R.J. Reynolds, Winston-Salem, NC, USA) were obtained from local commercial retailers. At day 29, rats were subdivided and given water as control, two doses of DOPs (50 mg/kg and 200 mg/kg b.wt.), or NAC (300 mg/kg b.wt., as positive control) daily via oral gavage for the remaining duration (Figure 1(b)). On day 57, rats were sacrificed 24 h after last exposure with overdose of pentobarbital (100 mg/kg; i.p.). Bronchoalveolar lavage (BAL) was obtained through washing with 1.5 ml ice-cold PBS for three times in total. After centrifugation at 1000 rpm for 10 min at 4°C to remove cellular debris, the supernatant was frozen in aliquots for further use. The largest lobe of the left lung tissue was fixed in 4% formalin solution and embedded in paraffin for sectioning. The remaining lung tissues were collected and frozen for further analysis. All animal procedures were performed in strict accordance with the guidelines from ARRIVE and Directive 2010/63/EU of the European Parliament, and the animal protocols were approved by the Committee on the Use of Live Animals in Teaching and Research (CULATR) of The University of Hong Kong (No. 4538-17).

### 2.5. RNA Extraction and RT-PCR for MUC5AC mRNA

Well-differentiated HBECs and lung tissues were harvested for RNA extraction using TRIzol reagent (Invitrogen, USA). Total RNA was DNase treated (Invitrogen, USA) before cDNA synthesis. Reverse transcription of RNA was conducted with EvoScript Universal cDNA Master kit (Roche, Basel, Switzerland) following the manufacturer's instruction. Quantitative RT-PCR assay was performed using SYBR Green Real-Time Master Mix (Applied Biosystems, Lithuania) based on the manufacturer's protocol. Relative quantity of mRNA was obtained by using the comparative *Ct* method and normalized by housekeeping genes such as human ribosomal protein S13 (RPS13) or rat glyceraldehyde 3-phosphate dehydrogenase (GAPDH). The primer for human MUC5AC is as follows: 5′-CTT TGG CAT CTG TGA GGA GC-3′ (forward primer) and 5′-CAC AGA AGC AGA GGT CTT GC-3′ (reverse primer). The primer for rat MUC5AC is as follows: 5′-AAC TCT GCC CAC CAC AAG C-3′ (forward primer) and 5′-TGC CAT CTA TCC AAT CAG TCC AAT-3′ (reverse primer).

### 2.6. MUC5AC ELISA

The method of MUC5AC ELISA was adapted from Parker et al.'s previous report [18]. Apical washing from the well-differentiated HBECs and BAL from rats was diluted (1 : 5 and 1 : 10, respectively) in carbonate-bicarbonate coating buffer (Sigma-Aldrich, St. Louis, MO, USA) and incubated at 37°C for 18 h. After washing with PBS/Tween-20 (0.05%), the plate was blocked with 2% BSA (Sigma-Aldrich) for 1 h at room temperature. After washing, a 1 : 200 dilution of MUC5AC mouse Mab (45M1; Thermo Fisher Scientific, Carlsbad, CA, USA) was added and incubated for 1 h at room temperature. A 1 : 2000 dilution of rabbit anti-mouse IgG antibody conjugated to HRP (Novus Biologicals, Littleton, CO, USA) was added after washing and incubated for 1 h at room temperature. After washing, tetramethylbenzidine (TMB) substrate solution (BD Biosciences, San Diego, CA, USA) was added and developed in the dark for 15 min. The reaction was stopped by adding 2N H_2_SO_4_, and the plate was read at 450 nm with 570 nm for wavelength correction using a spectrophotometer (CLARIOstar®, BMG Labtech, Ortenberg, Germany).

### 2.7. Mucus Viscosity

Apical washing from well-differentiated HBECs and BAL from rat was measured using a Brookfield LVDV-II+Pro cone and plate digital viscometer with CP-40 spindle (Brookfield AMETEK, MA, USA). The measurements were carried out at 25°C using a water bath with temperature controller. Viscosity measured at a speed of 40 rpm and shear rate of 300 1/s was used to compare the groups after normalizing to relevant control, adapted from Sánchez-Véliz et al.'s previous report [19]. Data were collected from the first 30 s of the experiments.

### 2.8. Transmission Electron Microscopy (TEM)

Selected ALI cultures of well-differentiated HBECs and rat trachea were fixed in 2.5% glutaraldehyde (Electron Microscopy Sciences, Ft. Washington, PA, USA) and sent to Electron Microscope Unit (EMU) of The University of Hong Kong for further processes. The samples were observed under a Philips CM 100 transmission electron microscope.

### 2.9. Histopathology

Alcian Blue/Periodic Acid-Schiff (AB/PAS) staining was applied to identify goblet cells in the airways. Images of 5 fields for epithelium in rat cartilaginous airways were captured randomly at ×40 magnifications by using a Nikon microscope (Nikon Instruments Inc., Tokyo, Japan) with a Nikon DS-Ri2 Digital Camera. The PAS-positive cells were measured using the software Nikon NIS-Elements BR.

### 2.10. Western Blot Analysis

Total protein of rat lung homogenate was separated in SDS-polyacrylamide gel and transferred onto a nitrocellulose membrane (GE Healthcare, Germany). After blocking, target protein was detected using specified antibody for analysis of phospho-EGFR (T678; ImmunoWay Biotechnology, Plano, TX, USA; 1 : 1000 dilution). Protein expression levels were normalized with *α*-tubulin.

### 2.11. Statistical Analysis

Data were presented as mean ± SEM. One-way ANOVA test with post hoc analysis (Bonferroni) was applied to compare multiple groups. Student's *t*-test was also carried out for variables measured at a single time point where appropriate. All the statistical analyses were performed using GraphPad Prism 7 (GraphPad Software Inc., San Diego, CA, USA). Significance was achieved if *p* value < 0.05.

## 3. Results and Discussion

**Figure 2:**
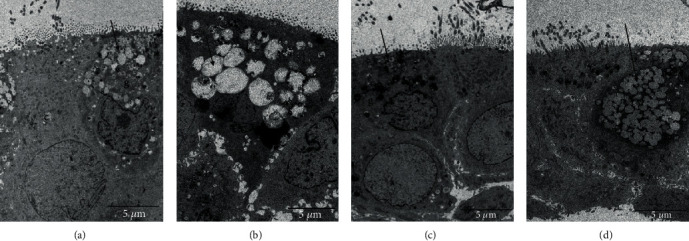
Transmission electron microscopy (TEM) images of *in vitro* model. Primary normal human bronchial epithelial cells (HBECs) after 28-day air-liquid interface (ALI) culture were stimulated with (a) control medium, (b) 4% cigarette smoke medium (CSM), (c) 4% CSM with *Dendrobium officinale* polysaccharides (DOPs) (1 *μ*g/ml), and (d) 4% CSM with N-acetylcysteine (NAC) (10 mM). c: ciliated cells; g: goblet cells; arrow: secretory vesicles. Scale bar: 5 *μ*m.

**Figure 3:**
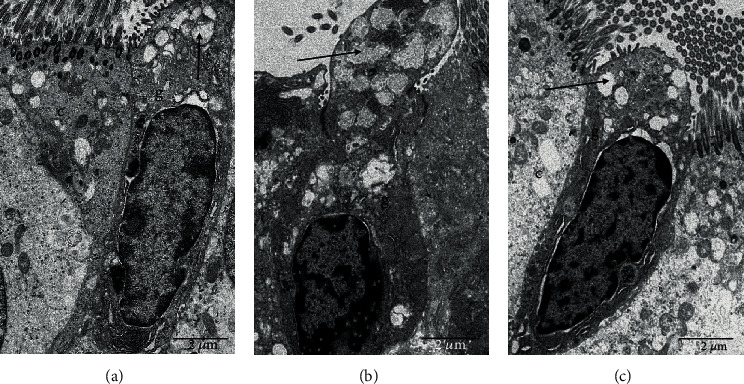
Transmission electron microscopy (TEM) images of airway epithelium of rat trachea. Male Sprague-Dawley (SD) rats were exposed to sham air (as control) or cigarette smoke for 56 days. At day 29, rats were subdivided as (a) sham air with water (as control), (b) cigarette smoke with water, and (c) cigarette smoke with DOPs (200 mg/kg b.wt.) via oral gavage for the remaining duration. c: ciliated cells; g: goblet cells; arrow: secretory vesicles. Scale bar: 2 *μ*m.

Under TEM, CSM or CS exposure caused an increase in the number of secretory vesicles and enlargement of the secretory vesicles associated with mucus secretion in the goblet cells *in vitro* (Figures 2(a) and 2(b)) and *in vivo* (Figures 3(a) and 3(b)), respectively, which were reversed by DOP treatment (Figures 2(c) and 3(c)). However, NAC treatment attenuated only the CSM-induced swollen secretory vesicles and caused no effect on the number of secretory vesicles (Figure 2(d)). The enlargement of secretory vesicles might lead to increased mucus secretion and viscosity. Treatment of DOPs diminished the number and the size of the secretory vesicles in both CS-exposed cell and rat models. As a mucolytic drug, NAC ameliorated only CS-induced swollen secretary vesicles in the goblet cells with no reduction in the number of secretory vesicles, suggesting the differential mucolytic effect between DOPs and NAC.

**Figure 4:**
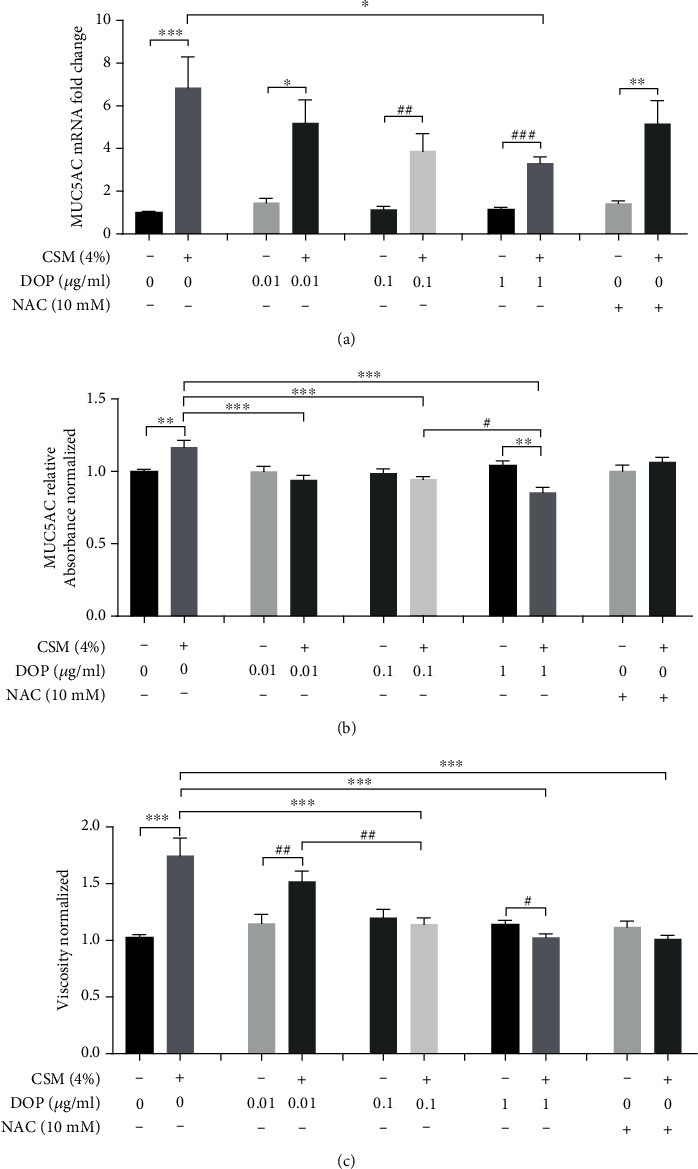
Effect of DOPs on CS-induced mucus secretion and viscosity in *in vitro* model. Primary normal human bronchial epithelial cells (HBECs) after 28-day air-liquid interface (ALI) culture were treated with DOPs or N-acetylcysteine (NAC) and then 4% cigarette smoke medium (CSM) for 24 hours. (a) Analysis of MUC5AC mRNA expression was carried out by real-time qPCR. (b) MUC5AC protein was quantitated by ELISA in apical washing. (c) Mucus viscosity was measured using a Brookfield LVDV-II+Pro cone and plate digital viscometer with CP-40 spindle in apical washing. Values are expressed as mean ± SEM (*n* = 5). ^∗^*p* < 0.05, ^∗∗^*p* < 0.01, and ^∗∗∗^*p* < 0.001 for one-way ANOVA test with post hoc analysis (Bonferroni). ^#^*p* < 0.05, ^##^*p* < 0.01, and ^###^*p* < 0.001 for Student's *t*-test.

**Figure 5:**
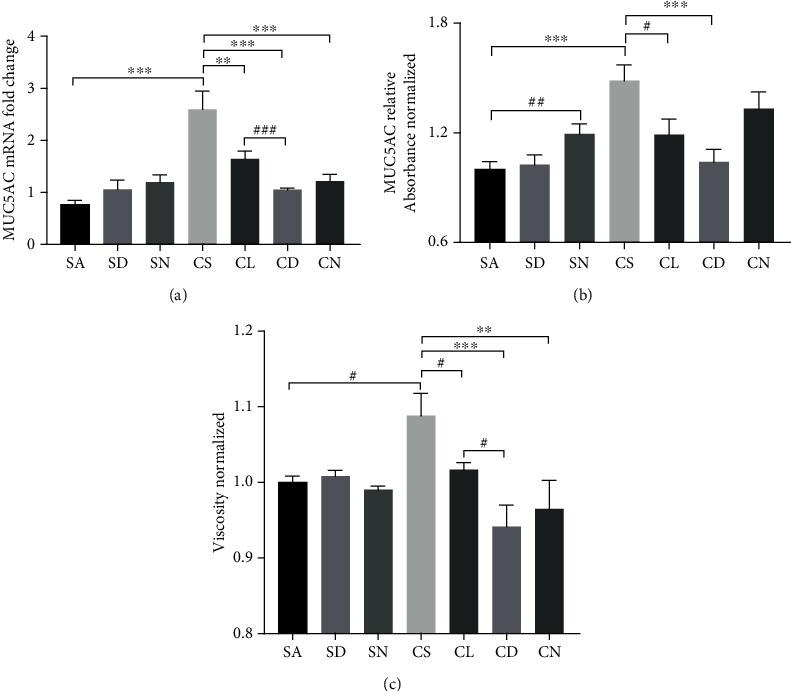
Effect of DOPs on CS-induced mucus secretion and viscosity in rat lungs. Male Sprague-Dawley rats were randomized to sham air (SA) (as control) or cigarette smoke (CS) group for 56 days. At day 29, rats were subdivided and given water as control (SA and CS groups), two doses of DOPs (100 mg/kg b.wt. and 200 mg/kg b.wt.) (SD, CL, and CD groups), or N-acetylcysteine (NAC; 500 mg/kg b.wt. as positive control) (SN and CN groups) daily via oral gavage for the remaining duration. (a) Analysis of lung MUC5AC mRNA expression was carried out by real-time qPCR. (b) MUC5AC protein was quantitated by ELISA in bronchoalveolar lavage (BAL). (c) Mucus viscosity was measured using a Brookfield LVDV-II+Pro cone and plate digital viscometer with CP-40 spindle in BAL. Values are expressed as mean ± SEM (*n* = 8). ^∗∗^*p* < 0.01 and ^∗∗∗^*p* < 0.001 for one-way ANOVA test with post hoc analysis (Bonferroni). ^#^*p* < 0.05, ^##^*p* < 0.01, and ^###^*p* < 0.001 for Student's *t*-test.

**Figure 6:**
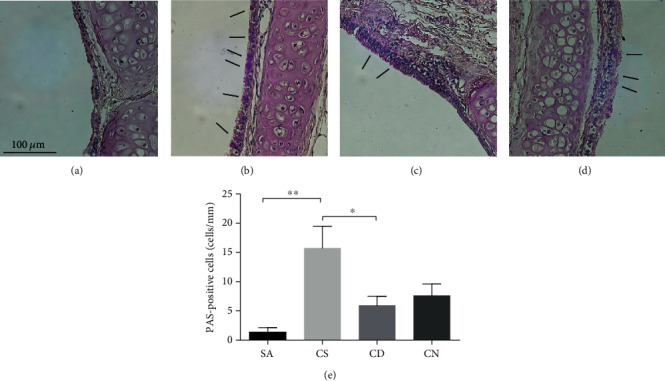
Effect of DOPs on CS-induced goblet cell hyperplasia in rat lungs. Male Sprague-Dawley (SD) rats were exposed to sham air (as control) or cigarette smoke for 56 days. At day 29, rats were subdivided as (a) SA: sham air with water (as control); (b) CS: cigarette smoke with water; (c) CD: cigarette smoke with DOPs (200 mg/kg b.wt.); and (d) CN: cigarette smoke with NAC (500 mg/kg b.wt.) via oral gavage daily for the remaining duration. (a–d) Representative images of cartilaginous airways in the rat lung sections stained with Alcian Blue/Periodic Acid-Schiff (AB/PAS). Scale bar = 100 *μ*m. Goblet cells appear as purple staining (arrows) over epithelium. AB/PAS staining revealed increased goblet cell hyperplasia after CS exposure and DOPs or NAC reduced the CS-induced goblet cell hyperplasia. (e) Quantification of AB/PAS-positive cells per length of epithelium for goblet cells of different groups. Values are expressed as mean ± SEM (*n* = 8). ^∗^*p* < 0.05 and ^∗∗^*p* < 0.01 for one-way ANOVA test with post hoc analysis (Bonferroni).

**Figure 7:**
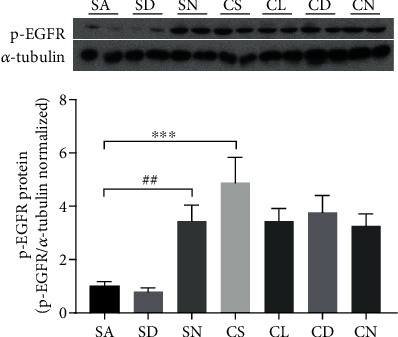
Effect of DOPs on CS-induced activation of epidermal growth factor receptor (EGFR) in rat lungs. Total protein from rat lungs was extracted. Phospho-EGFR was normalized by *α*-tubulin (housekeeping). Values are expressed as mean ± SEM (*n* = 8). ^∗∗∗^*p* < 0.001 for one-way ANOVA test with post hoc analysis (Bonferroni). ^##^*p* < 0.01 for Student's *t*-test.

CSM or CS exposure caused significant upregulation of MUC5AC mRNA expression in well-differentiated HBECs under ALI culture (Figure 4(a)) and in rat lung (Figure 5(a)), which was attenuated by DOPs in a dose-dependent manner. However, NAC had no attenuation of CSM-induced MUC5AC mRNA *in vitro* (Figure 4(a)) but caused significant reduction in CS-induced MUC5AC mRNA in rat lung (Figure 5(a)). Following a similar trend to gene expression, CSM or CS stimulated mucus hypersecretion in apical washing of well-differentiated HBECs under ALI culture (Figure 4(b)) and in rat BAL (Figure 5(b)) by MUC5AC ELISA, which was effectively reversed by DOPs dose dependently (Figures 4(b) and 5(b)). NAC had no effect on CSM- or CS-induced mucus hypersecretion *in vitro* and *in vivo*. CSM or CS induced significant elevation of mucus viscosity in apical washing (Figure 4(c)) and in rat BAL (Figure 5(c)), respectively, which was ameliorated by DOPs and NAC (Figures 4(c) and 5(c)). In line with the *in vivo* findings, CS exposure caused a significant increase in the number of goblet cells containing mucus in the epithelium of the cartilaginous airways, which was reduced by the treatment with DOPs using PAS staining (Figure 6). Furthermore, previous findings suggested that epidermal growth factor receptor (EGFR) is essential to mucin synthesis in response to CS stimulation [12, 20]. CS exposure caused significant upregulation of phospho-EGFR protein expression in rat lung, which was not reversed by either DOPs or NAC (Figure 7). However, NAC alone caused elevation of phospho-EGFR protein expression in rat lung (Figure 7).

The findings of the present study for the first time showed that DOPs could ameliorate mucus hypersecretion and mucus viscosity via inhibition of swollen secretory vehicles in the airway epithelium in both CSM- or CS-exposed models *in vitro* and *in vivo*. Mucus is essential for its role in protecting the airways. However, chronic inflammatory lung diseases, such as COPD, are often associated with excessive mucus production, especially in chronic bronchitis. Smoking is a common stimulus to promote mucus secretion via synthesis and secretion of MUC5AC [21]. MUC5AC has been recognized as the predominant secretary mucin in human airway epithelial cells, and its expression increases in smokers and COPD patients [22]. In this study, CS exposure caused upregulation of mucin MUC5AC in both *in vitro* and *in vivo* models, in agreement with previous studies [11, 23]. DOPs inhibited CS-induced MUC5AC overproduction in *in vitro* and *in vivo* models, in line with Song et al.'s previous report [17]. However, NAC had no significant effect on CS-induced MUC5AC overproduction *in vitro* and *in vivo*. The mechanism of NAC as a mucolytic drug might work through depolymerization of the mucin glycoprotein oligomer by hydrolyzing the mucin disulfide bonds and led to lessening of mucus viscosity [14, 24]. Although previous findings suggested that NAC could reduce mucin MUCA5C expression [25], the current data provided evidence for the insufficiency of NAC as a mucolytic drug especially under healthy condition [26]. The present findings on DOPs suggest a potential effective drug for amelioration of mucus hypersecretion. It is well recognized that CS induces mucus viscosity, which is consistent with the current findings in both *in vitro* and *in vivo* models [27]. DOPs attenuated CS-induced mucus viscosity dose dependently in both cultured cells and rat models, while NAC effectively reduced CS-induced mucus viscosity only.

To further study the mechanism of cigarette smoke-induced MUC5AC overexpression in airway epithelium, the expression of EGFR, which plays a crucial role in mucin production [28], was investigated. Oxidant stimuli such as CS exposure activate the expression of EGFR, which leads to activation of MAP kinases resulting in mucin gene (MUC5AC) transcription and induces mucus hypersecretion [12, 28]. Consistent to the previous findings, CS caused elevation of activated EGFR expression in rat lung [20, 23]. However, DOP had no significant effect on the inhibition of CS-induced EGFR activation, indicating that the alternative pathways might be involved in manipulating mucin MUC5AC overproduction. Unexpectedly, NAC alone caused upregulation of activated EGFR in rat lung of the sham air-exposed group. Except as a mucolytic drug, NAC also acts as an antioxidant to overcome oxidant-antioxidant imbalance in the inflammatory airways [29, 30], suggesting that NAC may serve as an oxidant under healthy condition.

Nevertheless, the present study has certain limitations. Firstly, direct CS exposure was not applied on the apical side of the epithelium in the *in vitro* study to mimic the human settings. Secondly, a passive smoking rat model was used *in vivo*, which might not exactly reflect the condition of COPD patients who have actively smoked for a long time. Lastly, consolidating evidence for the mechanism of action of DOPs on viscosity is still lacking, which warrants further study.

In summary, DOPs produced its effective effects on CS-induced mucus hypersecretion and viscosity via the inhibition of the mucus secretory granules. These findings could be a starting point for considering the potential role of DOPs in the management of the smoking-mediated COPD. However, further research is needed.

## Data Availability

Data will be available on request (judithmak@hku.hk).

## Abbreviations

AB/PAS: Alcian Blue/Periodic Acid-Schiff
ALI: Air-liquid interface
BAL: Bronchoalveolar lavage
BPE: Bovine pituitary extract
BSA: Bovine serum albumin
COPD: Chronic obstructive pulmonary disease
CS: Cigarette smoke
CSM: Cigarette smoke medium
CULATR: Committee on the Use of Live Animals in Teaching and Research
DOPs: *Dendrobium officinale* polysaccharides
EGFR: Epidermal growth factor receptor
EMU: Electron Microscope Unit
GAPDH: Glyceraldehyde 3-phosphate dehydrogenase
HBECs: Human bronchial epithelial cells
hEGF: Human epidermal growth factor
HPLC: High-performance liquid chromatography
MAP: Mitogen-activated protein
MUC5AC: Mucin-5AC
NAC: N-Acetylcysteine
PBS: Phosphate-buffered saline
RT-PCR: Real-time polymerase chain reaction
RPS13: Ribosomal protein S13
SA: Sham air
SD: Sprague-Dawley
SEM: Standard error of the mean
TEM: Transmission electron microscopy
TMB: Tetramethylbenzidine
TPM: Total particulate matter.

## Supplementary Materials

Supplementary Materials Supplementary Table: examples of *Dendrobium*-based drugs and health food products approved by the State Food and Drug Administration in China. Supplementary Figure: HPLC fingerprint for total *Dendrobium officinale* polysaccharides (DOPs) used in this study.
