# Supplementary material for: Amelioration of Cigarette Smoke-Induced Mucus Hypersecretion and Viscosity by Dendrobium officinale Polysaccharides In Vitro and In Vivo
Source: Oxid Med Cell Longev. 2020 Oct 21;2020:8217642. doi: 10.1155/2020/8217642 (PMC7596542; doi:10.1155/2020/8217642)
Supplement: Supplementary Materials — Supplementary Table: examples of Dendrobium-based drugs and health food products approved by the State Food and Drug Administration in China. Supplementary Figure: HPLC fingerprint for total Dendrobium officinale polysaccharides (DOPs) used in this study. [file 8217642.f1.docx]

**Amelioration of cigarette smoke-induced mucus hypersecretion and viscosity by *Dendrobium* *officinale* polysaccharides *in vitro* and *in vivo***

Rui Chen^1^, Yingmin Liang^1^, Mary Sau Man Ip^1^, Kalin Yanbo Zhang^2^, Judith Choi Wo Mak^1,3,*^

Departments of ^1^Medicine and ^3^Pharmacology & Pharmacy and ^2^School of Chinese Medicine, Li Ka Shing Faculty of Medicine, The University of Hong Kong, Hong Kong SAR, CHINA

* Corresponding author:

Judith C.W. Mak, Departments of Medicine and Pharmacology & Pharmacy, Li Ka Shing Faculty of Medicine, The University of Hong Kong,

L8-40 Laboratory Block, 21 Sassoon Road, Pokfulam, Hong Kong

Tel: (+852) 39179753

Fax: (+852) 28162095

Email: [judithmak@hku.hk](mailto:judithmak@hku.hk)

**Supplementary Table.** Examples of Dendrobium-based drugs and health food products approved by the State Food and Drug Administration in China.

| Name | Function |
| --- | --- |
| Shihu Yeguang Wan  石斛夜光丸 | Replenish yin of the kidney; quench liver-fire; improve eyesight |
| Shihu Mingmu Wan  石斛明目丸 | Replenish yin of the kidney; quench liver-fire; improve eyesight |
| Fufang Shihu Pill  复方石斛片 | Replenish yin of the kidney; quench liver-fire; improve eyesight |
| Shihu Yeguang Granules  石斛夜光颗粒 | Replenish yin of the kidney; quench liver-fire; improve eyesight |
| Compound Fresh Shihu Granules  复方鲜石斛颗粒 | Replenish yin of the stomach; promotes the production of fluids |
| Dendrobium Officinale Granules  铁皮石斛颗粒 | Immune regulation |
| Dendrobium Officinale Tablets  铁皮石斛含片 | Immunity enhancement; throat clearing |
| Dendrobium Officinale Capsules  铁皮石斛胶囊 | Anti-fatigue; immunity enhancement |
| Tiepi Fengdou Granules  铁皮枫斗胶囊 | Anti-fatigue; immunity enhancement |

**Supplementary Figure. HPLC fingerprint for total Dendrobium officinale polysaccharides (DOPs).** A: not hydrolyzed; B: hydrolyzed, 1): mannose; 2): galactose; 3): arabinose; 4): xylose.
